# *Lymnaea palustris* and *Lymnaea fuscus* are potential but uncommon intermediate hosts of *Fasciola hepatica* in Sweden

**DOI:** 10.1186/1756-3305-6-251

**Published:** 2013-08-29

**Authors:** Adam Novobilský, Martin Kašný, Luboš Beran, Daniel Rondelaud, Johan Höglund

**Affiliations:** 1 Department of Biomedical Sciences and Veterinary Public Health, Section for Parasitology, Swedish University of Agricultural Sciences (SLU), Uppsala 750 07, Sweden; 2 Department of Parasitology, Faculty of Science, Charles University, Viničná 7, Prague 2 128 44, Czech Republic; 3 Department of Botany and Zoology, Faculty of Science, Masaryk University, Kotlářská 2, Brno 611 37, Czech Republic; 4 Agency for Nature Conservation and Landscape Protection of the Czech Republic, Kokořínsko Protected Landscape Area Administration, Česká 149, Mělník 276 01, Czech Republic; 5 INSERM U 1094, Faculty of Medicine and Faculty of Pharmacy, 2 rue du Docteur Raymond Marcland, Limoges 87025, France

**Keywords:** *Fasciola hepatica*, *Fascioloides*, *Galba truncatula*, Intermediate host, ITS-2, *Lymnaea fuscus*, *Lymnaea palustris*, Lymnaeidae, Metacercariae, Shell size

## Abstract

**Background:**

*Lymnaea palustris* and *L. fuscus* are members of the European stagnicolines (Gastropoda: Lymnaeidae). The role of stagnicolines in transmission of *Fasciola hepatica* has been often proposed. To assess the possible relationship between these two stagnicolines and *F. hepatica* in Sweden, field monitoring in parallel with experimental infections of *L. palustris* and *L. fuscus* were conducted.

**Methods:**

Stagnicoline snails were collected and identified on pastures grazed by either sheep or cattle on four farms suffering from fasciolosis in Sweden during 2011–2012. Field-collected *L. palustris* and *L. fuscus* were examined for *F. hepatica* DNA by PCR. In the laboratory, different age groups of *L. palustris, L. fuscus* and *G. truncatula* were each exposed to two *F. hepatica* miracidia and main infection characteristics were obtained.

**Results:**

One field-collected *L. palustris* (out of n = 668) contained *F. hepatica* as determined by PCR. On the other hand, stagnicolines artificially exposed to *F. hepatica* miracidia resulted in successful infection with fully differentiated cercariae, but only in juvenile snails (size, 1–2 mm at exposure) and with a prevalence of 51% and 13% in *L. palustris* and *L. fuscus*, respectively. In contrast, 90% of juvenile (size, 1–2 mm) and 92% of preadult *G. truncatula* (size, ≥ 2-4 mm), respectively, were successfully infected. Delayed, reduced and/or no spontaneous cercarial shedding was observed in the two stagnicolines when compared to *G. truncatula*. However, at snail dissection most cercariae from *L. fuscus* and *L. palustris* were able to encyst similarly to those from *G. truncatula*.

**Conclusion:**

Both *L. fuscus* and *L. palustris* can sustain larval development of *F. hepatica* but with an apparent level of age resistance. The finding of a single *F. hepatica* positive specimen of *L. palustris*, together with infection characteristics from the experimental infection, suggest that *L. palustris* is a more suitable snail vector of *F. hepatica* than *L. fuscus*. The reduced growth observed in both stagnicolines was contrary to the ‘parasitic gigantism’ theory. Overall, it seems that the epidemiological role of *L. palustris* in transmission of *F. hepatica* in Sweden is likely to be much lower than for *G. truncatula*.

## Background

The liver fluke *Fasciola hepatica* is a worldwide distributed generalistic parasite with a di-heteroxenous life cycle including both mammalian (mainly ruminants) and certain gastropod hosts [1]. Several snail species within the family Lymnaeidae play an essential role as intermediate hosts for this digenean. In Europe, *Galba truncatula* is considered to be the principal intermediate host of *F. hepatica* in most environments [2]. However, in some locations it has been shown that that it can be replaced by other taxonomically related snails, such as *Omphiscola glabra*[3] and *Radix* spp. [4,5]. Although *G. truncatula* is considered the main intermediate host of *F. hepatica* in Scandinavia, this has so far been confirmed only in Denmark [6].

The phylogeny and generic classification of the family Lymnaeidae is a problematic issue, with divergent conclusions between studies [7]. A morphologically similar group of snails within the family Lymnaeidae, commonly known as stagnicolines [8], has been suggested to be potentially susceptible to *F. hepatica*[9-11]. According to recent phylogenetic studies, there is evidence for a clear genetic variability between European and North American stagnicolines. Five formerly recognized European stagnicolines (*Stagnicola palustris*, *S. turricula*, *S. fuscus*, *S. corvus*, *S. occulta*) [12,13] have all recently been reclassified [10,11] into four taxa as follows: *Lymnaea palustris* (formerly *S. palustris* and *S. turricula*), *L. fuscus*, *L. corvus*, *Catascopia terebra* (formerly *S. occulta*) [10,14,15].^a^ All of these stagnicoline species, except *C. terebra*, are present in Sweden, where they occur in marshland and/or near lakes, and often share habitat with *G. truncatula*[16,17]. It may be hypothesized that all stagnicoline snails are exposed to *F. hepatica* miracidia in habitats grazed by *F. hepatica*-infected ruminants, and along with *G. truncatula* they may serve equally well as intermediate hosts for the parasite. The infection of *L. palustris* and *L. fuscus* with *F. hepatica* miracidia has been completed under laboratory conditions [18,19], but these data need to be further confirmed, especially due to the above-mentioned taxonomic problems within the family Lymnaeidae. Since knowledge about the infection status of European stagnicoline snails is limited, the main aim of this study was to determine the potential of *L. palustris* and *L. fuscus* as intermediate snail hosts in the transmission of *F. hepatica.*

## Methods

### Detection of *F. hepatica* in field-collected snails

#### Study areas and snail collection

To screen for the potential vector capacity of stagnicolines in Sweden, snails collected on three beef cattle farms and one sheep farm were monitored during a two year period. During the first visits in May and June 2011, potential snail habitats were identified and a survey for infected snails was initiated. All four farms had a history with fasciolosis and prevalence in slaughtered animals exceeding 50%. Snail habitats (sampling sites) at all three cattle farms were marshy coastal areas near freshwater lakes and were represented by a gradual transition between the lake and the grazing area. At the sheep farm (Lilla Edet), the snail habitats consisted of drainage ditches and creeks localized between improved pastures. Beside stagnicolines, *G. truncatula* was also present at all farms. While the habitats of stagnicolines and *G. truncatula* completely overlap, and snails were located in the mud (no water layer) at the three beef cattle farms, a biotope boundary line was apparent at Lilla Edet. Thus, stagnicoline populations at the three beef cattle farms possess a high level of amphibiousity, commonly known only for *G. truncatula*. In contrast, the habitat (deeper water bodies) of the stagnicoline population at Lilla Edet was similar to those described for *Radix* spp. or *Lymnaea stagnalis*[13].

Snail collection was always performed by the same person and in the same quadrates during all visits, at 20 min time intervals in accordance with a previous study [20]. After their collection, snails were placed in 0.1 L plastic containers with water and then transferred to the laboratory. Within 24 hours of collection, all snails were classified according to their shell morphology [12,13] and individually frozen (−20°C) in microfuge tubes for further examination.

#### Morphological and molecular identification of snails

Four to ten snails per population (farm), representing each ecotype and sharing the same shell morphology, were euthanized in hot water and placed in 70% ethanol for morphological determination of their genitalia. These snails were identified on the basis of the length of their reproductive organs and the shape of the bursa copulatrix according to taxonomic keys [12,13].

Furthermore, two additional snails per population showing the same morphology as mentioned above were selected for molecular identification. This was based on amplification and sequencing of the internal transcribed spacer 2 (ITS-2) region of the ribosomal DNA [11]. Briefly, genomic DNA was extracted from whole snail bodies (2 specimens per farm) using DNeasy Blood and Tissue Kit (Qiagen, Germany) according to the manufacturer’s instructions. Polymerase chain reaction (PCR) amplification of snail ITS-2 was carried out with primers designed in a previous study [11] (forward: LT1 5′-TCGTCTGTGTGAGGGTCG-3′; reverse: ITS-2 Rixo R: 5′-TTCTATGCTTAAATTCAGGGG-3′), in 25 μl reaction tubes, as follows: 1 μl of snail DNA, 10 mM Tris–HCl pH 8.3, 50 mM KCl, 2 mM MgCl_2_, 4 μg Bovine Serum Albumin (BSA; New England Biolabs, Inc., UK), 0.8 μM of each primer, 0.2 mM dNTP and 0.3 U Taq polymerase (Ampli Taq Gold, Applied Biosystems, USA). Separation, purification and sequencing of the PCR products was carried as described earlier [21]. Sequences were edited with CLC Main Workbench (version 5.6.1), and compared with sequences available in GenBank (accessed in March 2013) [GenBank: AJ243017; AJ272051; AJ296271; AJ319620; AJ319621; AJ319622; AJ319623; AJ319624; FR797838; FR797839; FR797840; FR797841; FR797845; FR797846; FR797847; FR797848; HE577631; HE577632; HE577633; HE577634; HE577635; HE577636; HE577637; HE613327; JN614443; JN614444; JN614445; JN614446; JN614447; JN614448; JN614449], using Clustal W2 (EMBL-EBI) and the basic local alignment search (BLAST) tool (http://blast.ncbi.nlm.nih.gov/).

#### Monitoring of snail infection by PCR

Field-collected snails (size range, 8–18 mm) were thawed and examined by a PCR method designed to detect *F. hepatica* larval DNA as follows. Snails were examined as pooled samples (pooled after the lysis step of DNA extraction) that consist of 2–10 individual snails per sample. Whole snail bodies were individually crushed with sterile pestles and mechanically disrupted. Tissue was lysed in 20 μl of proteinase K and 180 μl lysis buffer (Qiagen, Germany) at 56°C overnight. After lysis, 20 μl of each lysed individual were taken into a pooled sample. Genomic DNA was extracted from each pool using the DNeasy Blood and Tissue Kit as described earlier. Sensitivity of *F. hepatica* PCR was verified by prior testing of a pool containing the lysate from 9 uninfected snails and 1 experimentally infected snail. Extracted pooled snail DNA was then amplified by PCR using Ampli Taq Gold kit. *F. hepatica* specific primers (forward: FH-ITS2-SPEC-F 5′-CTTATGATTTCTGGGATAATT-3′, reverse: FH-ITS2-SPEC-R 5′-CCGTCGCTATATGAAAA-3′) described in [22] were used to amplify 112 bp region of the *F. hepatica* ITS-2 gene. The PCR mixture was prepared in the identical proportions, as described earlier for PCR amplification of ITS-2. Amplifications were generated in a 2720 Thermal Cycler (Applied Biosystems, USA), by 40 cycles of 45 sec at 95°C, 1 min at 55°C and 1 min at 72°C, preceded by 10 min at 95°C and followed by 5 min at 72°C. The amplification products were separated on 1.5% agarose gel stained with GelRed™ Nucleic Acid Gel Stain (Biotium, USA). Whenever *F. hepatica* positive pools samples were identified all individuals in the same sample were re-tested according to the above mentioned PCR protocol. To eliminate any possible error of morphological species determination for any infected individual snails, their identity was also verified by sequencing as stated above.

The specificity of primers was evaluated by testing DNA samples originating from adult *F. hepatica*, adult *Fascioloides magna*, *L. palustris* experimentally infected with *F. hepatica* or *F. magna* and one specimen of *G. truncatula* naturally infected with *Haplometra cylindracea*. The specificity was compared with a previously described protocol [5,23] based on amplifying of a specific fragment of the cytochrome C oxidase subunit 1 gene (*cox*-1) of *F. hepatica*.

### Experimental infections of snails

#### Parasite

Eggs of *F. hepatica* were isolated from bovine livers by washing bile ducts at an abattoir in Skövde (western Sweden). *F. hepatica* eggs were washed several times with physiological saline, and stored in a 0.3 cm tap water layer at 4°C in the dark until use. To initialize embryonation, suspended eggs were incubated for 15 days at 25°C in the dark. Hatching was stimulated by exposing the mature eggs to intensive light (approx. 4000 lux), and after 30 min miracidia attracted to the light source were collected.

#### Snails exposed to miracidia

To compare the susceptibility of stagnicoline species to *F. hepatica* and to characterize intramolluscan development of *F. hepatica*, different age and size groups of *L. fuscus*, *L. palustris* and *G. truncatula* (which was included as a susceptible reference) were exposed to *F. hepatica* miracidia. All three snail species were morphologically and molecularly identified to species as described above. In the case of *L. fuscus* and *L. palustris*, adult snails were collected from the field and then maintained in plastic containers in the laboratory for egg production. The F1 generations were hatched and then used for the study. The parent generations of the *L. fuscus* and *L. palustris* populations originated from Lilla Edet and Norrköping, respectively (Table 1). Specimens of *G. truncatula* were collected from a road ditch outside Uppsala, Sweden (coordinates: 59.88 N 17.58 E), and from an area where livestock and wildlife were excluded from grazing. A total of 200 juvenile *G. truncatula* (in the size range 1–4 mm) were collected and used in the experiment. Examination of 200 *G. truncatula* from the same site revealed no trematode infections. Snails of all three isolates were measured (Biltema digital caliper 16–105, Sweden; with an accuracy 0.03 mm), and divided into 16 experimental groups as shown in Table 2.

**Table 1:** Origin of snail populations collected from the field

			**Number of collected snails**		
**Locality**	**Coordinates**	**Type of farm**	***Lymnaea palustris***	***Lymnaea fuscus***	**GenBank accession number**
			**n = 668**	**n = 130**	
Norrköping	58.62 N, 16.38 E	beef cattle	323		[GenBank:KC248373]*
Linköping	58.46 N, 15.56 E	beef cattle	245		[GenBank:KC248374]
Kristianstad	56.07 N, 14.11 E	beef cattle	100		[GenBank:KC905167]
Lilla Edet	58.09 N, 12.23 E	sheep		130	[GenBank:KC248371]**

* F1 generation of this *L. palustris* isolate was used in the experimental study.

** F1 generation of this *L. fuscus* isolate was used in the experimental study.

**Table 2:** Summary of snail species and groups used in the experimental study

	**Shell size at day of exposure**					
	**1-2 mm**	**1-2 mm**	**≥ 2–4 mm**	**≥ 2–4 mm**	**≥ 4–6 mm**	**≥ 4–6 mm**
Snail species	***Lymnaea fuscus***					
Number of snails at exposure	100	50	100	50	100	50
Number of miracidia	2	–	2	–	2	–
Survival rate at day 30 post-exposure (%)	69	90	79	86	100	100
Mean shell height (±S.D.) of CS + NCS snails at their death (mm)	5.9 ± 1.6	11.2 ± 0.9	13.4 ± 0.9	13.8 ± 0.8	15.3 ± 1.1	15.1 ± 0.8
Snail species	***Lymnaea palustris***					
Number of snails at exposure	100	50	100	50	100	50
Number of miracidia	2	–	2	–	2	–
Survival rate at day 30 post-exposure (%)	78	82	91	90	100	100
Mean shell height (± S.D.) of CS + NCS snails at their death (mm)	6.8 ± 2.2	10.6 ± 0.8	12.9 ± 1.6	13.3 ± 1.3	14.1 ± 1.3	14.5 ± 1.6
Snail species	***Galba truncatula***					
Number of snails at exposure	100	50	100	50	–	–
Number of miracidia	2	–	2	–	–	–
Survival rate at day 30 post-exposure (%)	57	66	74	88	–	–
Mean shell height (± S.D.) of CS + NCS snails at their death (mm)	4.6 ± 1.0	4.5 ± 1.1	6.2 ± 1.2	5.8 ± 1.0	–	–

Abbreviations.

*CS* cercariae shedding snails (spontaneous shedding of cercariae during experiment).

*NCS* non cercariae shedding snails (snails that did not produce cercariae spontaneously but contained *F. hepatica* larvae).

#### Experimental protocol

Individual snails were exposed to 2 miracidia in a small volume (300 μl) of artificial pond water (APW) dispensed into the wells of a 96-well plastic plate. Miracidia were added to each well, closed with conical caps, and incubated for 12 hours. Following miracidial exposure, all snails of the same species and the same group were transferred to a plastic box (40 × 30 × 19 cm) containing a 4 cm deep layer of APW, where they were maintained during the first 30 days post-exposure (PE). All boxes were placed in an air-conditioned room at 22°C and with a photoperiod including a 10 hours light cycle. During the experiment, snails were fed *ad libitum* with pesticide-free fresh lettuce, and the APW was changed weekly. After 30 days PE, each surviving snail was transferred to a 3.5 cm Petri dish and maintained according to the same conditions as during the first 30 days. Petri dishes were checked daily for cercariae and/or metacercariae under a stereomicroscope at a magnification of 15×. Water was changed daily and ≈ 1 cm^2^ of decomposed lettuce was added. From day 50 PE for *G. truncatula* and day 60 PE for *L. fuscus* and *L. palustris*, all surviving snails in both the exposed and non-exposed groups were subjected to thermal shock [24] every second day, by placing their Petri dishes at 10°C for 3 hours to stimulate cercarial emergence. At day 65 PE for *G. truncatula* and at day 95 PE for *L. fuscus* and *L. palustris*, surviving snails were measured with a digital caliper. Snails were then crushed and their soft tissues were individually transferred into new Petri dishes with fresh water for cercarial shedding. Encysted and non-encysted cercariae were counted 2 hours after crushing.

### Testing of *in vitro* excystment of *F. hepatica* metacercariae

*In vitro* excystment was applied to test the viability and excystment ability [25] of *F. hepatica* metacercariae. These larvae were collected from all three snail species after shell crushing, and were maintained in water at 4°C in the dark for a maximum of one month. To remove the outer cyst a total of ≈ 100 metacercariae per snail species were exposed to 1% sodium hypochlorite by adding 2 ml of the salt solution to the microfuge tube for 20 min at 20°C. After incubation, metacercariae were washed 3 times with pond water and once with Activating and Excysting Medium (AEM). AEM consisted of RPMI-1640 medium (Sigma-Aldrich, USA), 1% antibiotic antimycotic solution (code A5955, Sigma-Aldrich, USA), 0.9% bovine bile (Oxoid, UK) and 0.006% L-cysteine (Sigma-Aldrich, USA). Metacercariae were then transferred into a 24-well plate (1 ml of AEM per well) and incubated at 38°C in the dark with a CO_2_ level of 20%. After 4 hours, newly hatched juveniles and non-excysted metacercariae were counted at a magnification of 200× under a stereomicroscope with transmitted light. Each batch of metacercariae originating from *L. fuscus*, *L. palustris*, and/or *G. truncatula* was tested in triplicate.

### Data and statistical analyses

In experimental infections, survival rate at day 30 PE and the prevalence of *F. hepatica* infection in relation to the number of surviving snails at day 30 PE were determined. The following characteristics were recorded: mean and total number of spontaneously emerged cercariae from cercariae-shedding (CS) snails; first day of cercarial shedding; mean number of metacercariae obtained from snails where no cercarial shedding (NCS) was observed until dissection; and shell size at the end of the experiment. All statistical analyses were performed using GraphPad Prism 5.02 (GraphPad Software, USA). Analysis of variance (ANOVA) was applied to assess differences between mean sizes of infected snails, exposed but uninfected snails, and the snails from the negative control group. ANOVA, followed by Bonferonni post-hoc tests, were also applied to compare differences in metacercarial production. Pearson correlation analysis was carried out to evaluate the relationship between shell size and cercarial production. All statistical tests were considered significant when p ≤ 0.05.

## Results

### Snail identification

The anatomical structure of the reproductive organs, especially the length of the preputium and the shape of the bursa copulatrix at section, in the stagnicoline isolate from Lilla Edet agreed with those already reported for *Stagnicola fuscus*[12,13]. Also, the ITS-2 sequence of the Lilla Edet isolate [GenBank: KC248371] had 98–99% similarity to *Lymnaea fuscus* genotypes 1 and 2 [GenBank: AJ319622; AJ319621] and *Stagnicola fuscus* [GenBank: HE577637; HE613327; HE577636; HE577634; HE577633]. Furthermore, the snail isolates collected from Norrköping, Linköping and Kristianstad were morphologically identical to *L. palustris*[12,26]. Following a BLAST search of ITS-2, all three isolates [GenBank: KC248373; KC248374; KC905167] had 99% similarity to *L. palustris* or *L. turricula* [GenBank: JN614468; AJ319620; AJ457043; HQ283267; AJ319618; AJ319619; HE577632; HE577631; FR797839; FR797838; FR797840; FR797841]. The sequence of the Uppsala isolate of *G. truncatula* [GenBank: KC248372] used in the experimental study had 100% identity to French and Venezuelan isolates of *G. truncatula* [GenBank: JN614444; JN614445; JN614443] [10]. For quantitative proportion of collected snails see Table 1.

### Specificity of primers

**Figure 1:**
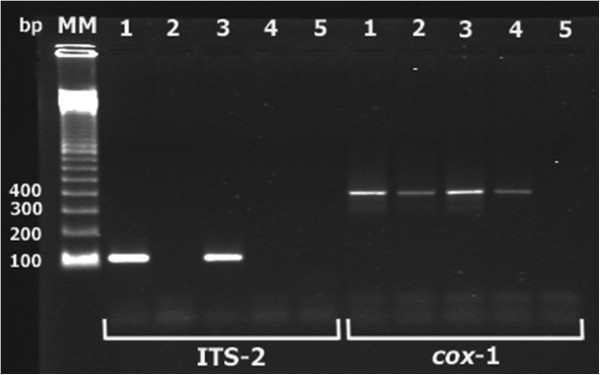
**Agarose gel electrophoresis of amplified ITS-2 and *cox*-1; evaluation of the specificity of our PCR method and the method previously described **[23]**: 1**. adult *Fasciola hepatica*; 2. adult *Fascioloides magna*; 3. *Lymnaea palustris* experimentally infected with *F. hepatica*; 4. *L. palustris* experimentally infected with *F. magna*; 5. *Galba truncatula* naturally infected with *Haplometra cylindracea*. The first five (1–5) PCR products (ITS-2) were obtained according to our described ITS-2 gene amplification method (from the left side); five others (*cox*-1) were obtained according to the previously used PCR protocol with primers for mitochondrial cytochromoxidase 1 [23].

After PCR with ITS-2 region specific primers (described in [22]) and DNA isolated from adult *F. hepatica* and *L. palustris* snails (experimentally infected with *F. hepatica*) followed by agarose electrophoresis the 112 bp PCR product was recorded. No visible amplicons were observed after control PCR with DNA samples isolated from adult *F. magna*, neither snails infected with *F. magna* and/or *H. cylindracea*. In the PCR with *cox*-1 region specific primers (described in [23]), however, PCR products (≈405 bp) for analyzed samples (DNA from *F. hepatica*, *F. magna*, *L. palustris*) were recorded (Figure 1).

### Monitoring of *F. hepatica* infection of snails collected in the field

A total of 668 snails determined as *L. palustris* [GenBank: KC248373; KC248374; KC905167] from all three sampling sites (Norrköping, Linköping and Kristianstad) during 2011–2012 were examined for liver fluke DNA by PCR. Only one *L. palustris* collected on the beef farm in Linköping was *F. hepatica* positive. The species identity of this positive specimen was confirmed by morphology and ITS-2 sequencing [GenBank: KC248374]. All *L. fuscus* snails (n = 130) were negative.

### Experimental infection of snails

In both *L. fuscus* and *L. palustris* no infected snail was observed in the ≥ 2–4 mm or ≥ 4–6 mm groups, and only juvenile snails (1–2 mm) were infected, with respective prevalences of 13% and 51% (Table 3). The survival of snails 30 days PE in all three snail isolates was improved with increasing shell size. In both size groups of *G. truncatula* and in the 1–2 mm groups of both *L. palustris* and *L. fuscus*, survival rates were significantly lower in infected snails than in the negative controls (Table 2). All results from the experimental infections are summarized in Table 3. In juvenile *L. fuscus*, only one snail spontaneously shed cercariae (18 metacercariae, 68^th^ day PE). In addition, seven snails were successfully infected but they did not produce any cercariae until dissection. In juvenile *L. palustris*, 17 (of 40) *F. hepatica* positive snails spontaneously shed cercariae from 61 days PE. Most snails in the 1–2 mm and ≥ 2-4 mm groups of *G. truncatula* were infected (respective prevalences, 90% and 92%), and more than 50% also spontaneously shed cercariae from day 43 PE. Significant differences between the stagnicoline groups and *G. truncatula* were noted for the spontaneous cercarial shedding, and also for metacercarial counts obtained from NCS snails, with the highest values in preadult (≥ 2-4 mm) *G. truncatula* and the lowest in juvenile *L. fuscus*.

**Table 3:** **Infection characteristics of *Lymnaea fuscus, Lymnaea palustris *and *Galba truncatula***

	**Snail species**			
**Infection characteristic**	***Lymnaea fuscus***	***Lymnaea palustris***	***Galba truncatula***	
Shell size at day of exposure (mm)	1-2	1-2	1-2	≥ 2-4
Prevalence of *F. hepatica* infection (%)	13.0	51.3	89.5	91.9
Number of infected snails (CS + NCS)	9	40	51	68
Number of snails with cercarial shedding (CS)/snails without cercarial shedding (NCS)	1/8	17/23	34/17	59/9
First observed cercarial shedding (day post exposure)	68	61	46	43
Mean number of spontaneously shed cercariae per CS snail ± S.D.	18^a^	18 ± 23^a^	103 ± 80^b^	130 ± 102^b^
Total number of metacercariae in all CS snails	18	348	3619	7952
Mean number of MC per NCS snail (± S.D.) at dissection (after crushing)	157 ± 58^a^	165 ± 51^a^	269 ± 114^b^	299 ± 88^b^

Superscript letters (a, b) indicate significant differences (p < 0.05) in metacercarial/cercarial production between groups.

CS, cercariae shedding snails (spontaneous shedding of cercariae during experiment).

NCS, non cercariae shedding snails (snails that did not produce cercariae spontaneously but contained *F. hepatica* larvae).

**Figure 2:**
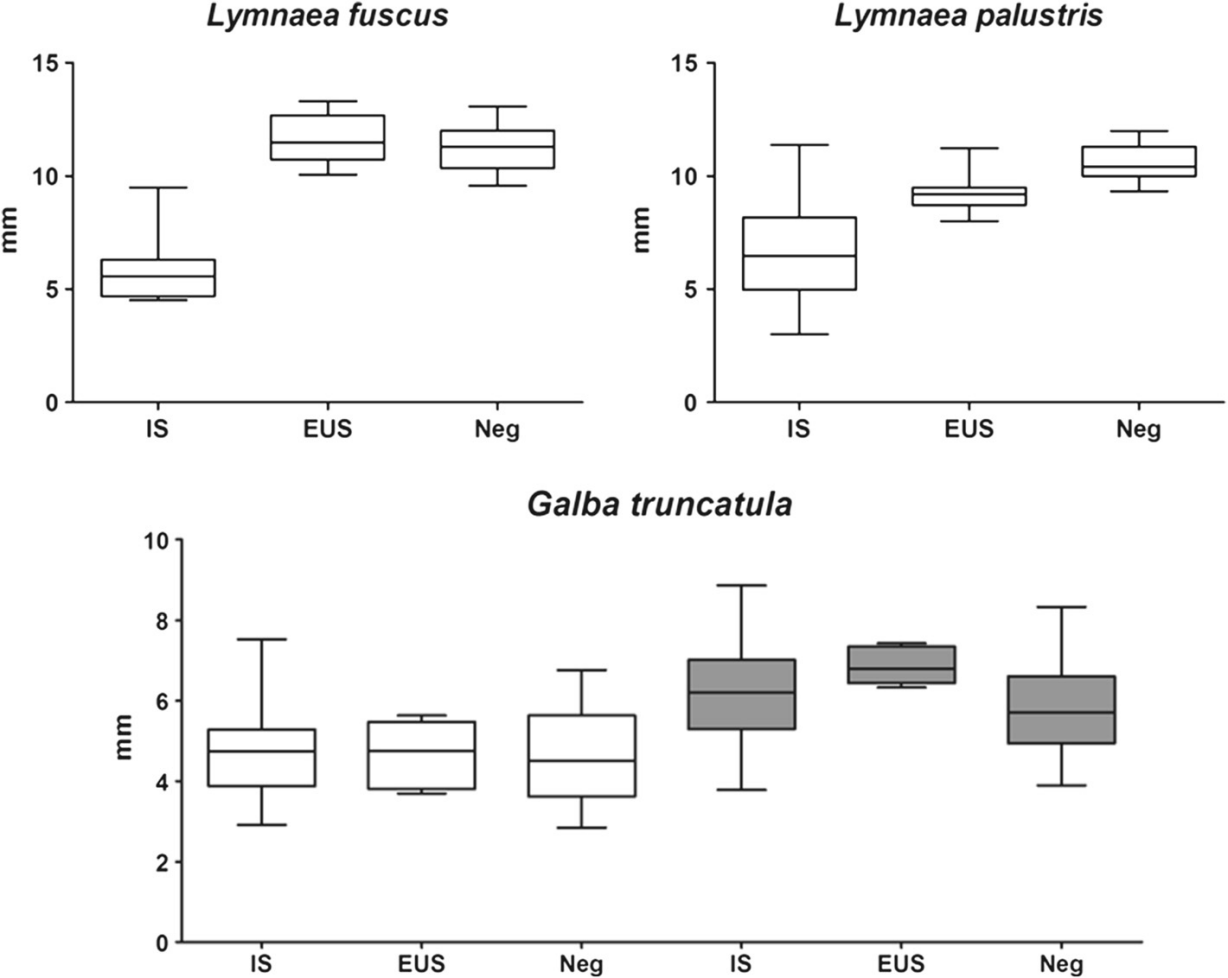
**The effect of *Fasciola hepatica *infection on the size of *Lymnaea fuscus*, *L. palustris *and *Galba truncatula*.** The data are expressed as mean shell sizes of groups 1–2 mm for *L. fuscus* and *L. palustris* (measured at day 95 PE) and groups 1–2 mm (white) and ≥2-4 mm (grey) for *G. truncatula* (measured at day 65 PE). The horizontal line inside each box represents the arithmetic mean of each group; whiskers are standard deviations. Abbreviations: IS, infected snails (cercarial shedding or no cercarial shedding); EUS, snails exposed but uninfected; Neg, negative controls.

Significant positive correlations were found between shell size and the production of metacercariae in *G. truncatula* (1–2 mm group: r^2^ = 0.54, p < 0.0001; ≥ 2–4 mm group: r^2^ = 0.28, p < 0.0001), but not in the stagnicoline species. However, shell size at the end of the experiment was significantly (p < 0.0001) lower in infected snails than in the negative group for both stagnicoline species, whereas it did not differ between infected and uninfected *G. truncatula* (Figure 2).

The *in vitro* excystment rate of metacercariae obtained from NCS snails after crushing was 18%, 23% and 79% for *L. fuscus*, *L. palustris* and *G. truncatula*, respectively.

## Discussion

Evaluation of the susceptibility of potential intermediate hosts to *F. hepatica* infection and exploration of the epidemiological importance of *Fasciola* sp. snail host requires a combination of approaches, such as field screening, experimental infection of snails and taxonomic determination of species (snails, parasites) also by using the molecular methods (PCR, sequencing, BLAST). Several studies refereed on susceptibility of lymnaeid snail hosts to *F. hepatica*[4,5,18,19,27], but only few publications showed combined methodology which was documented for *Radix balthica* and *R. labiata*[4]. Our study, therefore, brings the first combined approach in evaluation of vectorial capacity of European stagnicoline snails and their potential role in epidemiology of *F. hepatica*.

Although *L. fuscus* and *L. palustris* were previously considered to be low susceptible (for juveniles measuring 2 mm or less in height at miracidial exposure) or resistant (the older snails) to *F. hepatica* infection [28], both of these stagnicoline species have occasionally been listed as intermediate hosts [11,29]. In this study, we have scrutinized the stagnicoline - *F. hepatica* relationship both by detailed investigation of snails collected from the field and by confirmatory experimental infections, including comparison to parallel infection of the most typical intermediate host for *F. hepatica* in Europe, i.e. *G. truncatula*. In our study we compared the intramolluscan development of *F. hepatica* in the two stagnicolines with that of *G. truncatula*; it can be concluded that although the presence of parasite DNA in the snail bodies was confirmed, these stagnicolines must be regarded as accidental hosts of *F. hepatica*, and their ecological role in the transmission of the parasite is probably minor. On the other hand, as it has been suggested for *Radix* spp. [4,5] the role of *L. palustris* as a vector might be more important in *G. truncatula* free areas.

Owing to inherent taxonomic problems with the morphological identification of stagnicoline snails [8,11], molecular genetic methods have become essential to avoid confusion [8,10,11]. Snail conchology is not always conclusive and may sometimes result in false species identification [10,11]. In addition, adults of *G. truncatula* can easily be mistaken with pre-adult *L. palustris* or *L. fuscus* if the determination is based only on shell morphology. The agreement between ITS-2 sequences and morphological determination (length proportions of male copulatory organs and shape of bursa copulatrix) further confirms that the assessment of snail internal anatomy is an applicable method for lymnaeid species identification [13,30].

Examination of *F. hepatica* in snails by dissection and morphological determination of rediae and cercariae constitute the classical screening method [3,20,31-33]. However, a PCR-based surveillance test for *F. hepatica* in snails was proposed from the 1990s [34]. When our newly applied PCR protocol based on the amplification of a specific fragment within the ITS-2 region was validated for the related *F. magna*[22], it showed high specificity when both DNA of adults and snails experimentally infected with *F. magna* were tested. In contrast, amplification of *cox-1*[23] resulted in cross-reaction between *F. hepatica* and *F. magna* (Figure 1). These findings show that the PCR method, including those primers applied in the present study, can be used in the future as a specific diagnostic tool to distinguish between *Fasciola* spp. and *Fascioloides*.

With this PCR-based method only one snail (out of 668 *L. palustris*) was found to be *F. hepatica* positive. Although this finding is insignificant from an epidemiologic point of view, it demonstrates that *L. palustris* can be infected with *F. hepatica* under natural field conditions. However, since snails were examined only by PCR, we cannot say whether the infection was patent or not. To our knowledge, the only cases of natural infection of *L. palustris* with *F. hepatica* were reported from Central France [35] and also in the east of this country in a single specimen [31]. Since *L. fuscus* was found only at one farm and the total number of examined snails was lower than that of *L. palustris*, we cannot fully exclude natural infection of this species elsewhere.

Data from the experimental study clearly show that both *L. palustris* and *L. fuscus* are susceptible to *F. hepatica* and that the parasite can complete its development in both species. However, in comparison to *G. truncatula* these stagnicoline snails were harder to infect and there was also clear evidence of age resistance. Lower survival of infected snails, when compared to that noted in the negative control groups, coincides with reports on this or various other trematode and snail species [21,36-38]. Increased parasite-induced mortality in juvenile snails infected with trematodes is common [39], and is usually related to i) mechanical alteration of tissues by penetration of miracidia and/or ii) the growth and migration of first-generation rediae (mother rediae) [40]. Suppressive effect of trematode infection on immune system, nutrition of juvenile snails and general snail fitness is also known in other snail species [41]. This effect of snail survival was, moreover, supported by the fact that survival rates between the exposed and negative groups did not vary in older categories of *L. palustris* and *L. fuscus* (≥ 2-4 mm, ≥ 4-6 mm), where snails were exposed but remained uninfected.

The absence of *F. hepatica* infection in both stagnicoline snails with shell size greater than 2 mm at exposure and high prevalence in both age groups of *G. truncatula* suggest that 2 mm constitutes the onset of age resistance in *L. palustris* and *L. fuscus* to *F. hepatica*. The age resistance in snails has already been reported for *L. palustris*[9,42] and *L. fuscus*[19,21], and it is, therefore, legitimately considered as one of the important markers of snail susceptibility to *F. hepatica* infection [9]. A possible explanation for age resistance might be related to the development of immune mechanisms during the first days after snail hatching, as has been documented earlier in *L. palustris*[42]*.* Thus, natural resistance of *L. palustris* against *F. hepatica* was explained by the fact that miracidia are able to penetrate into the snail body but forming sporocysts are immediately encapsulated and removed by the snail defense mechanisms [42]. Our results almost coincide with those of prior studies [28,42], but it seems that development of the snail immune mechanism may last up to 14 days according to our observations.

Metacercarial production and the time of first cercarial shedding uncover important details about the snail-trematode interaction. Whilst the highest reported metacercarial production in *G. truncatula* was 1789 metacercariae in a snail exposed to 5 miracidia [43], the average metacercarial production under experimental conditions was 154 per snail after spontaneous shedding, and 344 after dissection 55 days post exposure (at 20°C) [44]. This is in agreement with the values obtained for *G. truncatula* in the present study. In both stagnicoline species, metacercarial production was significantly lower and also more delayed than in *G. truncatula.* This suggests that despite the successful differentiation of cercariae, the larval development process was obviously delayed. Furthermore, only a few specimens of *L. palustris* and a single *L. fuscus* shed cercariae spontaneously. Most cercariae from *L. fuscus* and *L. palustris* encysted in the Petri dish mainly at dissection (day 95 PE), and with an average production of 157–168 metacercariae per infected snail. This is in agreement with the very low spontaneous cercarial production observed in accidental intermediate hosts of *F. magna*, and also when fully differentiated cercariae were found at dissection [21]. This phenomenon is difficult to explain. Nevertheless, as in the current study, it reflects the fact that the studied snail-trematode interactions were suboptimal.

The effect of *F. hepatica* infection on snail growth is a frequently studied variable concerning snail-trematode interactions. Compared to the shell height of negative controls, a significantly reduced size (stunting) of infected snails was noted for both stagnicoline species. Furthermore, as no difference in growth between infected and uninfected *G. truncatula* was observed, this contradicts the ‘parasitic gigantism’ hypothesis observed in lymnaeid snails [45]. Although other studies observed increased growth in *F. hepatica*-infected *G. truncatula* when compared to uninfected controls [46,47], an inverse effect on shell size has been shown in other lymnaeids such as *L. palustris*[18], *L. fuscus*[19], *L. stagnalis*[18] and *Pseudosuccinea columella*[48]. As we already have suggested for *F. magna*[21], this reduction effect on growth in lymnaeids other than *G. truncatula* probably indicates the level of co-evolution, which gradually will happen when the parasite and snail live in close ecological interaction with one another. Obviously, this controversy in growth effect caused by *F. hepatica* needs further research. Interestingly, cercarial/metacercarial production in our study positively correlated with shell size of *G. truncatula* but not with that of stagnicoline snails. The dependency of cercariae amounts on snail volume has been reported in *G. truncatula* as well as in other lymnaeid species [49,50].

## Conclusions

From field investigations and experimental infections of snails, it is clear that both *L. fuscus* and *L. palustris* can sustain larval development of *F. hepatica* but with an apparent level of age resistance. Nevertheless, when all infection characteristics in both stagnicolines are compared, it was evident that *L. palustris* is better suited as an intermediate host, and thus more suitable as a vector of *F. hepatica* in Sweden than is *L. fuscus*. This is further supported by the finding of a naturally infected *L. palustris* in the field. In fact, the present finding represents the first case where species identification of both the stagnicoline snail and its parasite was confirmed by a molecular method and sequencing. Spontaneous cercarial shedding, viability of metacercariae in *L. palustris*, together with a high level of amphibiousity in its natural habitats make this species likely as an intermediate host of *F. hepatica* in Sweden, along with *G. truncatula*. However, because of age resistance, the role of *L. palustris* in transmission of the trematode in natural habitats is likely to be much lower than for *G. truncatula*. Further, the existence of ‘parasitic gigantism’ was doubted in this study. Thus, we believe that the effect on snail growth may be related to co-adaptation/co-evolution of the trematode and snail.

## Endnotes

^a^These taxonomic names are used throughout the text and the term ‘stagnicoline’ to describe all species with a similar shell morphology, including *Lymnaea palustris*, *L. fuscus*, *L. corvus*, and *Catascopia terebra*.

## Competing interests

The authors declare that they have no competing interests.

## Authors’ contributions

AN designed the study, participated in field collections, performed all laboratory work and wrote main parts of the manuscript. MK helped with writing of the manuscript and participated in molecular identification of snail species. LB carried out morphological identification of snails. DR helped with designing of experimental infections and made comments to the manuscript. JH conceived the study and attracted the funding, as well as contributed to the interpretation of the data and writing of the manuscript. All authors have read and approved the final manuscript.
